# Disentangling Fat and Sugar in the Western Diet: Parental Consumption of a High-Fat Diet Alters Offspring Brain Development

**DOI:** 10.3390/nu18213539

**Published:** 2026-10-27

**Authors:** Gail Lee, Karina Wilk, Taylor DeYoung, Cheryl Chong, Emma G. W. McKnight, Tiffany Ayoub, Lloyd Fan, Jacob Ellegood, Antoine Beauchamp, Jane A. Foster, Tie-Yuan Zhang, Jason P. Lerch, Mark R. Palmert, Brian J. Nieman

**Affiliations:** 1 Mouse Imaging Centre, The Hospital for Sick Children, Toronto, ON M5T 3H7, Canada; taylor.deyoung@sickkids.ca (T.D.); emma.mcknight@alumni.utoronto.ca (E.G.W.M.); tiffany.ayoub@alumni.utoronto.ca (T.A.); jason.lerch@ndcn.ox.ac.uk (J.P.L.); 2 Translational Medicine, The Hospital for Sick Children, Toronto, ON M5G 0A4, Canada; 3 Neuroscience and Mental Health, The Hospital for Sick Children, Toronto, ON M5G 0A4, Canada; karina.wilk@uwaterloo.ca; 4 Department of Medical Biophysics, University of Toronto, Toronto, ON M5G 1L7, Canada; jellegood@hollandbloorview.ca; 5 Department of Biochemistry, University of Waterloo, Waterloo, ON N2L 3G1, Canada; cheryl724chong@gmail.com; 6 Department of Medicine, McMaster University, Hamilton, ON L8S 4L8, Canada; lloydfan1@gmail.com; 7 Bloorview Research Institute, Holland Bloorview Kids Rehabilitation Hospital, Toronto, ON M4G 1R8, Canada; abeauchamp@hollandbloorview.ca; 8 Department of Psychiatry and Behavioural Neurosciences, McMaster University, Hamilton, ON L8S 4L8, Canada; jane.foster@utsouthwestern.edu; 9 Center for Depression Research and Clinical Care, Department of Psychiatry, UT Southwestern Medical Center, Dallas, TX 75247, USA; 10 Douglas Mental Health University Institute, Department of Psychiatry, McGill University, Montréal, QC H4H 1R3, Canada; tieyuan.zhang@douglas.mcgill.ca; 11 Wellcome Centre for Integrative Neuroimaging, University of Oxford, Oxford OX3 9DU, UK; 12 Department of Physiology, The University of Toronto, Toronto, ON M5S 1A8, Canada; mark.palmert@cw.bc.ca; 13 Division of Endocrinology, The Hospital for Sick Children, Toronto, ON M5G 1X8, Canada; 14 Division of Endocrinology, BC Children’s Hospital, Vancouver, BC V6H 3N1, Canada; 15 Ontario Institute for Cancer Research, Toronto, ON M5G 0A3, Canada

**Keywords:** neurological disorders, parental dietary patterns, neurodevelopment, magnetic resonance imaging, brain structure, gestational diet exposure, mouse models

## Abstract

Background: Maternal consumption of the Western diet is a precursor for metabolic conditions such as obesity and is associated with increased offspring risk for neurodevelopmental disorders (NDDs). It is not clear which component (i.e., high-fat or high-sugar) of the Western diet contributes to changes in offspring neurodevelopment. We used a mouse model of parental consumption of five different diets containing varying levels of fat and sugar to examine and compare their effects on offspring brain structure. Methods: Male and female offspring (avg *n* = 15/sex/diet) were assessed for differences in body weight and developmental milestone achievement, and their brains were imaged at postnatal day (P) 65 using magnetic resonance imaging to compare brain structure. Results: Offspring of the high-fat-only and combined high-fat/high-sugar diet groups showed delayed righting reflex success and heavier weight at weaning. Brain structure differences were also observed in early adulthood (P65), with the pattern of change induced by high-fat/high-sugar diets similar to that observed in some autism mouse models. Conclusions: Parental high-fat-only and combined high-fat/high-sugar diets altered offspring brain structure, but the high-sugar-only diet did not. High fat consumption is likely a key driver of the observed offspring brain changes and is a modifiable risk factor for NDDs.

## 1. Introduction

Neurodevelopmental disorders (NDDs), such as autism spectrum disorder (ASD) and attention-deficit/hyperactivity disorder (ADHD), affect approximately 4–18% of children and youth in North America [1,2]. Individuals with NDDs face challenges in academic life, social relationships, and/or employment [3,4,5,6]. Families and health care systems are also impacted [4,7]. While medication and therapy can reduce the impacts of NDDs, there are currently no cures [8], which raises the question of whether prevention is possible in some instances. One potentially modifiable risk factor for NDDs is maternal metabolic status, such as pre-pregnancy obesity, excessive gestational weight gain, and diabetes [9,10,11,12]. The continued rise in the rates of obesity, especially in people of child-bearing age [13], makes this risk factor particularly important.

Diet is a precursor leading to downstream metabolic conditions such as obesity, which are associated with NDD risk in offspring [14]. Rodent models enable the use of experimental diets in controlled environmental and genetic contexts to investigate the link between early-life diet exposures and brain development. This has revealed, for example, altered offspring social behaviour and synaptic potentiation linked to changes in gut microbiota after high-fat consumption in mice [15], and depression-like behaviour with changes in myelin-related gene expression in rat offspring after maternal high-fat diet exposure [16]. Developmental changes are also evident in offspring brain structure after parental high-fat or high-fat/high-sugar diet exposure [17,18], though structural changes are not evident after paternal exposure without maternal exposure [19]. Although many studies have used rodent models of high-fat diet consumption, human Western dietary patterns comprise both high-fat and high-sugar contents [20]. Studies have not systematically compared changes in brain structure outcomes in offspring following parental high-fat, high-sugar or combination exposures. Separating the high-fat versus high-sugar components and their different effects on the structural development of offspring brains is crucial in elucidating the potential mechanisms by which parental diets and downstream metabolic changes manifest and increase offspring NDD risk. Assessing offspring brain structural changes against established phenotypes in mouse models of NDDs may provide insight into whether diet-induced alterations reflect disease-relevant features.

In this study, our aim was to determine how components of the Western diet—fat, sugar or their combination—in parents affect offspring brain development as assessed by structural magnetic resonance imaging (MRI), which allows for a high-resolution and high-throughput characterization of changes in structure across the whole brain with broad relevance to brain function. We used C57BL/6J mice and provided them one of five different diets containing varying levels of fat and sugar for a 6-week acclimation period and then during breeding, gestation, and lactation. Our results indicate that parental diet fat content, in particular, alters the developmental trajectory of the brain in offspring in this mouse model and also suggest that the combination of high-fat and high-sugar in a parental diet more closely recapitulates structural phenotypes seen in ASD mouse models.

## 2. Materials and Methods

### 2.1. Diets and Animals

Five-week-old C57BL/6J mice were obtained from The Centre for Phenogenomics’s (TCP) in-house colony and kept on a 12 h light/dark cycle with access to food and water ad libitum (see Supplementary Table S1). The mice were assigned groups in a block-randomized fashion (2–5 breeding pairs per block) and placed on one of the following diets: (1) the control diet (CD); (2) the high-sugar diet (HS); (3) the high-fat/high-sugar diet (HFHS); (4) the high-fat 43 diet (HF43); or (5) the high-fat 60 diet (HF60). These mouse diets were chosen to enable variation of key components of the Western diet (i.e., high simple sugar and high fat content) [20] while maintaining similar content otherwise. Macronutrient content and diet identification details are provided in Table 1 (additional information is also available in Supplementary Table S2). Similar diets have been used in previous research [15,16,17] to produce weight gain and/or metabolic phenotypes comparable to humans [21]. Since the high-fat-content diets tended to crumble, all diet pellets were placed on the cage floor throughout the experimental time course. Due to evident visual differences in diets (colour and texture), investigators were not blinded to diet during experiments.

**Figure 1:**
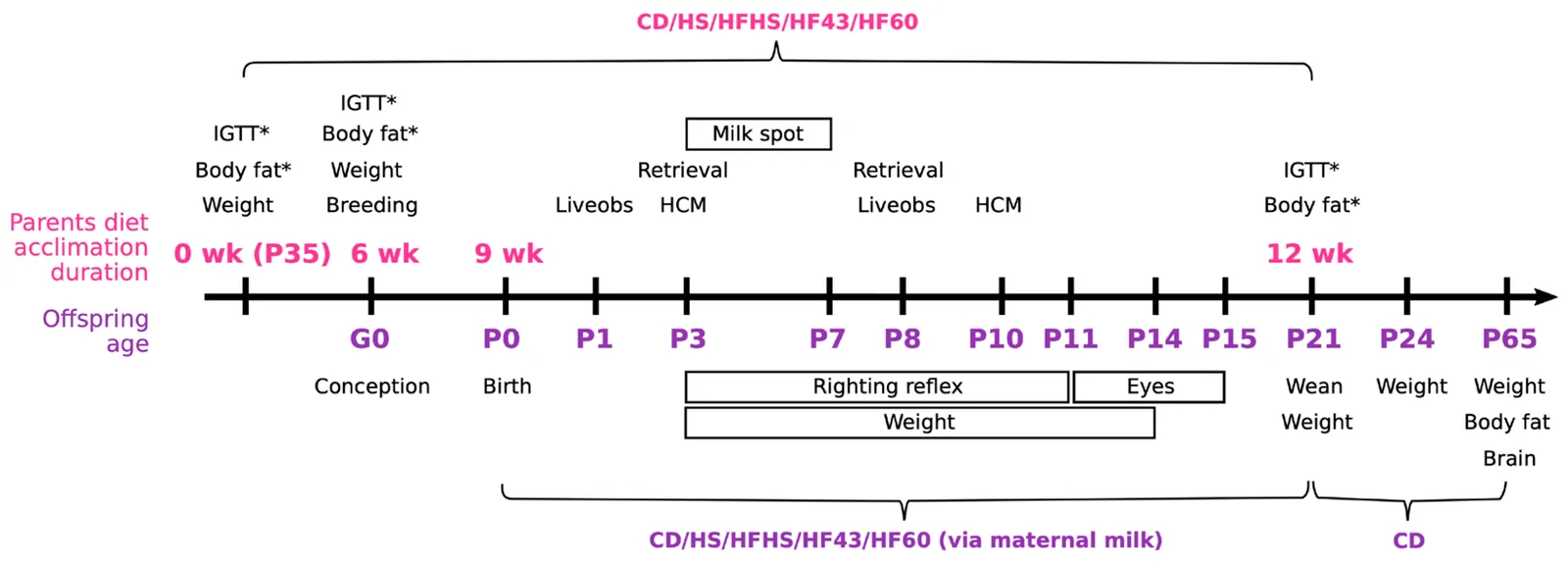
Experimental timeline. Pink colours above the horizontal line indicate parental diet acclimation duration, and purple colours below the line indicate offspring age. The measurements outlined in boxes were conducted once a day for the entire duration covered by the box. The measurements indicated by an asterisk were completed in a separate cohort of female mice. wk: week. G: gestational day. P: postnatal day. IGTT: intraperitoneal glucose tolerance test. Liveobs: live observation of maternal behaviour. HCM: home cage monitoring.

After a 6-week diet acclimation, which is sufficiently long to induce weight gain [17], male and female mice were paired for mating within their diet group for 4–5 days. Pregnant female mice were then singly housed and kept on their specified diet throughout gestation and lactation. At three weeks of age (i.e., postnatal day (P) 21), all offspring mice were weaned onto CD, regardless of parental diet, and remained on CD for the rest of the experiment (see Figure 1 for an experimental timeline). Parent mice were used for breeding only once to control the diet consumption period. Weaning to the CD allowed for assessment of long-term impacts of isolated perinatal diet exposure. At the time of weaning, pups weighing less than 7.0 g in any group were considered runts and excluded. The number of litters produced for each diet type were *n* = 10 CD, 10 HS, 8 HFHS, 11 HF43, and 8 HF60. The number of male (M) and female (F) offspring used for collection of experimental data was *n* = 19M/15F, 18M/15F, 14M/15F, 21M/15F, 13M/12F (CD, HS, HFHS, HF43, HF60). We aimed for a sample size of 15 mice per group per sex to provide sufficient power to capture ~3% volume change for main diet effects [22].

All animal care and experimental procedures were approved by the TCP Animal Care Committee (AUP 25-0175H).

### 2.2. Parental Physiology Observations

To evaluate the impact of diet on parental physiology, several observations of parents were collected (Figure 1). Dam and sire weights were measured before and after the 6-week diet acclimation period (CD 34 F/44 M; HS 25/25; HFHS 19/19; HF43 17; HF60 22/23). Additionally, in a separate cohort of females (not used for mating), glucose tolerance tests were performed at the start of the diet acclimation period and after the 6-week and 12-week exposure, corresponding to the time of breeding and the time of weaning, respectively, in the dams used for breeding. We chose a separate cohort of mice for these tests in order to avoid inducing stress in the dams and potential consequences for maternal behaviour or offspring neurodevelopment. This cohort included females only (owing to past results studying paternal effects [19]) and included 11 CD, 10 HS, 11 HFHS, 11 HF43, and 11 HF60. For this test, the mice were taken off the diets in the morning (between 9 AM and 12 PM) and fasted for 6 h (with access to water). After the 6 h fast, baseline blood glucose levels were measured from a tail poke using a glucometer (Accu-Chek^®^ Guide Glucose Meter, Roche, Indianapolis, IN, USA). Then, 10% glucose solution (G5767, Sigma-Aldrich, Oakville, ON, Canada) was administered via intraperitoneal injection (dose 0.01 mL/g), after which blood glucose levels were measured at the 30, 60, 90, and 120 min marks.

In the same cohort of female mice evaluated for glucose tolerance, an EchoMRI™-100H Body Composition Analyzer (EchoMRI™, Echo Medical Systems, Houston, TX, USA) was used to measure fat mass and lean mass in vivo [23] before and after diet acclimation. The measures provided by this test were fat mass, lean mass, free water, and total water. The body fat percentage was calculated as the percentage of fat mass over the total of fat and lean masses.

### 2.3. Maternal Behaviour Observations

To explore whether diet impacted maternal behaviour and care of pups, live observations of maternal behaviour were conducted in the morning on P3 and P10 (Figure 1). A total of ten 30 s observations were made for each dam, with 5 min inter-observation intervals. To avoid inducing stress, observations were made from outside of the cage without disturbing or moving the cage from its holding rack. Each observation was scored for the following seven maternal behaviours: active nursing, passive nursing, licking/grooming, nest building, nest attendance, nest nonattendance, and digging. Multiple behaviours within one 30 s observation window were permitted. The first five behaviours were classified as mothering behaviours, and the latter two were classified as non-mothering behaviours.

As the live observations were limited in duration, nest attendance over a 24 h period was also assessed as an indicator of mothering behaviour using a home cage monitoring system (Actual Analytics Ltd., Edinburgh, UK). This system consisted of eight enclosures, each of which can house a mouse cage and allows 24/7 video acquisition using an infrared camera. In the morning, each mouse cage was supplied with diet pellets and water before placement on the home cage monitoring rack. Monitoring occurred on P1 and P8. For scoring, the 24 h recording was edited into 10 s snippets extracted every 5 min over the entire 24 h, resulting in 287 snippets in total in a 48 min video. Each of the snippets was manually scored for dam location. If the dam was inside the nest for the full 10 s period, it was scored as inside the nest; otherwise, it was scored as outside the nest. An unexpected computer hardware failure resulted in loss of some data from the HS group.

Pup-directed maternal care was also evaluated through pup retrieval tests [24]. Four pups from the litter were placed on the far side of the cage from the nesting. The dam’s latency to retrieve the first pup and the total time to retrieve all 4 pups were measured. Nursing behaviour was also evaluated from ages P3 to P7 by observation of offspring milk spots. Observations were made in the mornings and scored as either 0 (milk spot absent) or 1 (milk spot present).

### 2.4. Offspring Milestones

Our previous data demonstrate that early-life diet exposure can lead to changes in adult brain structure [17]. To assess whether early-life diet alters early development, several milestones were assessed through the postnatal preweaning period (Figure 1). Offspring weights were measured from P3 to P14 and at P21, P24, and P65. Body fat percentage was measured at P65 using the EchoMRI™-100H Body Composition Analyzer.

From P3 to P11, offspring were evaluated with the righting reflex test in the mornings. One pup at a time was taken out of its cage and moved to an empty cage for the test. The pups were placed gently onto their backs and the time required for righting (all four paws touching the cage floor) was recorded. A maximum of 30 s was allowed, and any time exceeding this limit was considered a failure. The results were recorded as either a Fail or Success. After the 30 s test, the pups were rubbed with nesting material and put back into their home cage. After P6, if pups successfully righted themselves for 2 consecutive days, then they were not tested further.

From ages P11 to P15, offspring eyes were checked in the mornings and scored for opening: 0 for none, 0.5 for one eye, and 1 for two eyes open. Checks were stopped once both eyes of the pup were open.

### 2.5. Brain Sample Preparation, Image Acquisition, and Processing

To assess the neuroanatomy of the offspring, the offspring were sacrificed at a median age of P65 (range P64–P69) and fixed brain samples were prepared for magnetic resonance imaging, as previously described [25]. The mice were anesthetized with isoflurane (Fresenius Kabi, Friedberg, HE, Germany) and transcardially perfused with a 30 mL solution of 1 μL/mL heparin and 250 μL/mL gadolinium contrast agent (ProHance^®^, Bracco Imaging Canada, Ville D’Anjou, QC, Canada) in phosphate-buffered saline (PBS, 311-010-CL; Wisent Inc., Saint-Jean-Baptiste, QC, Canada). Then, they were infused with a 30 mL solution of 4% paraformaldehyde (PFA; Electron Microscopy Sciences, Hatfield, PA, USA) and 250 μL/mL Prohance in PBS for fixation, followed by decapitation and extraction of the skull. The brains were kept in the skull and placed in the same fixative solution of 4% PFA and Prohance for 24 h. The fixative solution was replaced with a storage solution comprising 250 μL/mL Prohance and sodium azide (Fisher BioReagents, Ottawa, ON, Canada) in PBS, and the brains were stored at 4 °C for a minimum of 1 month.

MR imaging was used to identify and quantify parental diet-induced neuroanatomical changes in the offspring at early adulthood. A multi-channel 7-Tesla 306 mm horizontal bore magnet (BioSpec 70/30 USR, Bruker, Ettlingen, Germany) with ParaVision 6.0.1 console software was used to acquire MR images for 8 brain samples simultaneously [26]. Brain anatomical images were obtained with a T2-weighted, 3D fast-spin echo sequence and the following parameters: TR = 350 ms, TE = 12 ms, 6 echoes, TEeff = 30 ms, field-of-view = 20 × 20 × 25 mm, matrix size = 504 × 504 × 630, 4 averages, total acquisition time = ~13 h, 40 µm isotropic resolution [26]. Diffusion tensor imaging was also performed (described in the Supplementary Materials).

For anatomical image processing, all images were registered together through the Pydpiper toolkit (version 2.0.18) [27], which utilizes the Advanced Normalization Tools (ANTs) [28] and the Montreal Neurological Institute (MNI) tools [29]. The Multiple Automatically Generated Templates (MAGeT) Brain Segmentation Algorithm [30] was used to segment each of the 157 mouse brain images into 185 bilateral brain regions using published atlases [31,32,33,34,35]. For this purpose, 25 images were randomly selected from the data set and mapped to the atlas. Each individual image in the data set was then registered to all 25 templates and the atlas to generate 26 candidate segmentations. In this work, volumes for each brain structure in the atlas were then estimated by computing the median volume across the 26 candidate segmentations.

### 2.6. Statistical Analysis

Statistical analysis was performed using R (version 3.6.3) through Rstudio (2022.07.1 + 554) [36,37]. Linear mixed-effects (LME) models were fit using the lme4 package (version 1.1.29) [38] with restricted maximum likelihood (REML). For analyses of maternal measurements and offspring development, fixed effects were included for diet, sex, age (factored, where measures were repeated), time (for the maternal glucose tolerance measures), and their interactions. Random effects were included for ID (for multiple measures in the same animal) or litter. A *p*-value < 0.05 was considered statistically significant. All *p*-values were computed using the Satterthwaite approximation for degrees of freedom [39].

For binary outcome variables (i.e., nest attendance in HCM and live observations, milk spots, righting reflex, eye opening), generalized linear models (GLM) were fit based on a binomial distribution [36]. GLMER was used to include random effects of ID for nest attendance in HCM. A *p*-value < 0.05, derived from the Wald test, was considered statistically significant.

In exploratory analyses of diet-induced global brain changes, a cluster analysis was conducted on all 185 brain structures to identify structures with similar patterns of volume change across diet groups, similar to the procedure in Yeung et al. [40]. First, each structure volume relative to the whole brain was fit with LME and the random effects of litter were subtracted from the volume. Then, the adjusted structure-wise relative volumes were z-scored and a vector was generated from the average values for each diet group, producing a matrix of 185 vectors each containing 5 values (for the 5 diets). The distance between the vectors was calculated using the Euclidean method, and hierarchical clustering was performed using the Ward D2 method [41]. The number of clusters retained was determined based on an elbow plot. The volume of each cluster (i.e., the summation of all member structures) was fit with a linear model to evaluate the significance of diet.

For analysis of individual structures, relative brain structure volumes (computed by normalizing to whole-brain volumes) were fit with an LME model, and fixed effects were included for diet (categorical), sex (categorical), and their interactions. A random effect was included that allowed for a litter-specific intercept. The false discovery rate (FDR) [42] was used to correct for multiple comparisons across all structures, and a *q* value < 0.1 was considered significant. Normality and homoscedasticity of volume data were assessed for each structure using the Kolmogorov–Smirnov and Fligner–Killeen tests, respectively, and found to be valid (adjusted *p*-values > 0.67 and 0.75, respectively). For the purpose of visualization, percent changes in brain structure volumes were calculated from the LME model by dividing the diet coefficient values by the coefficient of the intercept (representing the CD control group).

The mean and 95% confidence intervals reported in the text and displayed in figures were generated from the outputs of the model. For the offspring weights only, the 95% confidence intervals were calculated by bootstrapping 500 times with replacement. In the text, low and high 95% confidence intervals are indicated within square parentheses. 

### 2.7. Phenotype Comparison to ASD-Related Mouse Models

To evaluate the relevance of offspring brain phenotypes to mouse models of NDDs, the phenotypes were compared to a reference panel of 135 ASD-related mouse models, described fully in [43]. The anatomical phenotypes across the ASD models were previously clustered to generate related groupings, with 2 through 10 cluster solutions. Based on the genetics of grouped models and the Reactome database, molecular pathways enriched for each cluster grouping were reported. To explore ASD relevance and suggest molecular pathways of interest, the observed diet phenotypes were used to generate a voxelwise *t*-statistic map that was transformed to the space of the ASD panel. It was then used to compute the Spearman correlation with the median effect size maps of the ASD models. Best ASD cluster matches were identified for each diet group. Permutation of diet labels, respecting litter, was used to evaluate significance.

## 3. Results

### 3.1. Modified Diets Led to Weight and Metabolic Changes in Parent Mice and Induced Subtle Changes in Mothering Behaviour

**Figure 2:**
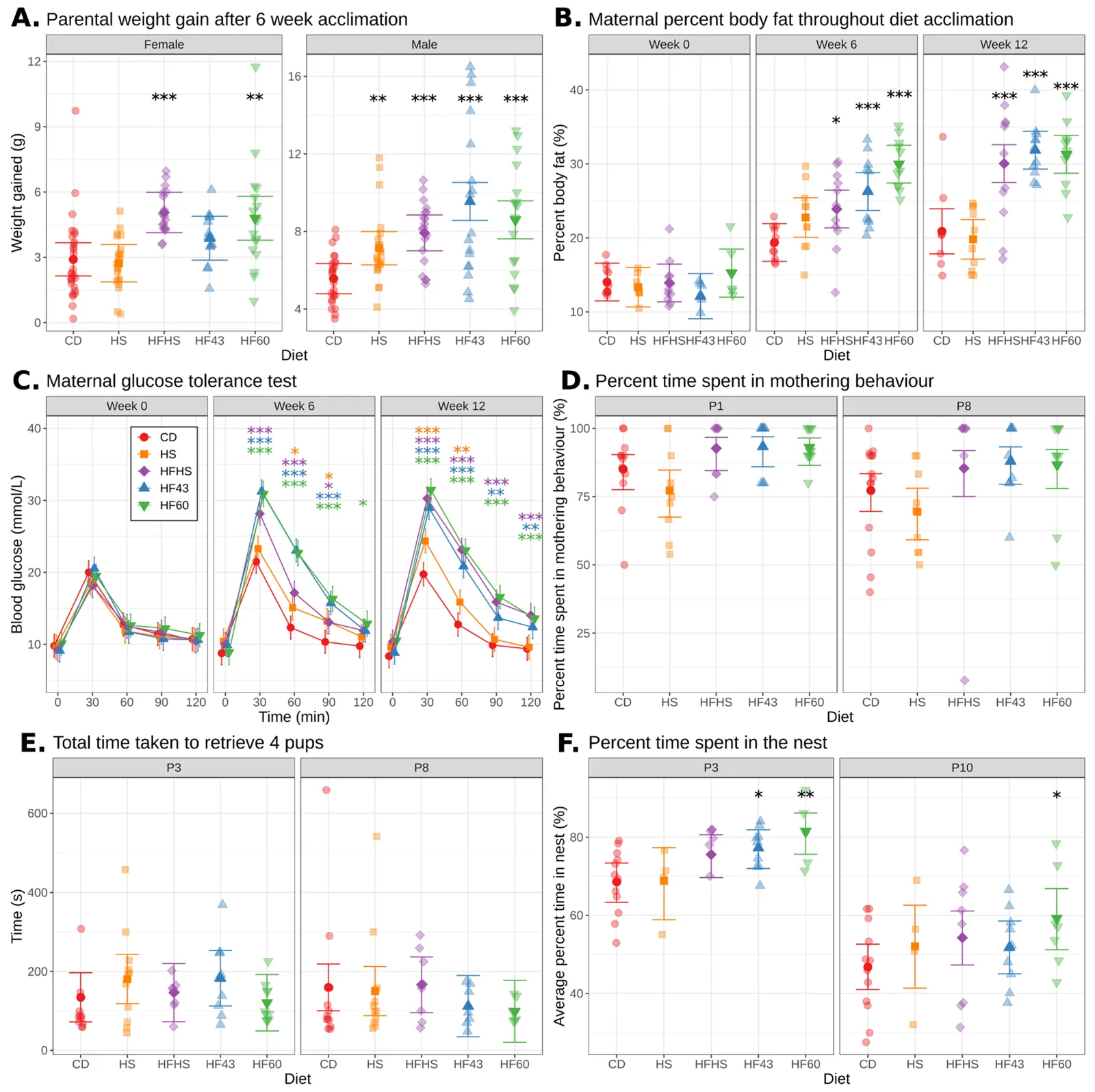
Parental weight, maternal metabolic, and offspring care evaluation measures. (**A**) Parental weight gain after 6 weeks of diet acclimation. (**B**) Maternal body fat percentage through 12 weeks of diet consumption. (**C**) Maternal glucose tolerance test. The *x*-axis represents the time since the intraperitoneal injection of the glucose solution. (**D**) Live observations of maternal behaviour. (**E**) Pup retrieval test. (**F**) Percent time spent in the nest over 24 hr home cage monitoring. The mean and 95% confidence intervals plotted were generated from the outputs of their corresponding models, and the individual data points represent individual mice. The data points have been jittered horizontally for visualization purposes. Asterisks denote: * *p* < 0.05; ** *p* < 0.01; *** *p* < 0.001.

The body weight, body composition, and glucose tolerance of parent mice were measured to verify the effects of diet manipulation. The dams on HFHS and HF60 showed significantly greater weight gain (5.1 g [4.1, 6.0] and 4.8 g [3.8, 5.8], respectively, *p* < 0.01) compared to CD dams (2.9 g [2.1, 3.7]), as did the sires on HFHS, HF43, and HF60 (7.9 g [7.0, 8.8], 9.5 g [8.6, 10.5], and 8.6 g [7.6, 9.6], respectively, *p* < 0.001) compared to CD sires (5.6 g [4.8, 6.3]; Figure 2A). The HFHS, HF43, and HF60 dams also showed elevated body fat percentage by the 6th week of diet acclimation (23.9% [21.3, 26.4], 26.3% [23.7, 28.8], and 30.0% [27.4, 32.5], respectively, *p* < 0.05) compared to CD dams (19.4% [16.8, 21.9]; Figure 2B). However, HS dams did not show a significant increase in weight gain (2.7 g [1.9, 3.6], *p* = 0.77) nor body fat percentage (at 6th week: 22.7% [20.1, 25.4], *p* = 0.08; at 12th week: 19.8% [17.1, 22.5], *p* = 0.60). The experimental diet dams also evidenced expected metabolic changes compared to CD mice in measures of glucose tolerance (Figure 2C): the dams consuming HFHS, HF43, and HF60 reached a peak blood glucose level of 28.2 mmol/L [26.6, 29.8], 31.2 mmol/L [29.6, 32.8], and 30.9 mmol/L [29.1, 32.6], respectively, 30 min after an IP injection of glucose solution by the 6th week of diet acclimation, which was higher than CD dams (21.5 mmol/L [19.9, 23.1], *p* < 10^−8^). By the 12th week, the HS mice also reached a higher peak of 24.4 mmol/L [22.7, 26.0] compared to CD dams (19.7 mmol/L [18.1, 21.3], *p* < 0.001). As early as the 6th week, all experimental diet-consuming dams also took longer to return to baseline and had an average blood glucose level of 14.6 mmol/L [13.7, 15.4] at 90 min post-injection, significantly higher than that of CD dams (10.3 mmol/L [8.7, 11.9], *p* < 0.05), indicating an impaired glucose tolerance.

The potential for diet-induced changes on mothering behaviour was evaluated. There was no significant difference between the experimental diet dams and CD dams in nest attendance time in maternal live observation (at P1: CD 85.1% [77.5, 90.4], HS 77.3% [67.5, 84.8], HFHS 92.7% [84.6, 96.7], HF43 93.3% [85.9, 97.0], and HF60 93.0% [86.5, 96.5], *p* > 0.06; Figure 2D) nor in total time taken to retrieve pups displaced from the nest (at P3: CD 134.3 s [71.9, 196.7], HS 180.5 s [118.2, 242.8], HFHS 146.3 s [72.6, 219.9], HF43 182.7 s [112.2, 253.1], and HF60 120.7 s [49.2, 192.3], *p* > 0.2; Figure 2E). However, in 24 h home cage monitoring at offspring age P3, the HF43 and HF60 dams spent more time in the nest (77.3% [72.0, 81.8] and 81.4% [75.6, 86.1], respectively) compared to CD dams (CD 68.6% [63.3, 73.4], *p* < 0.05; Figure 2F). There was no significant difference in the presence of offspring milk spots across diet groups (Supplementary Figure S1).

### 3.2. Perinatal Diet Alterations Affected Neonatal Developmental Milestones

**Figure 3:**
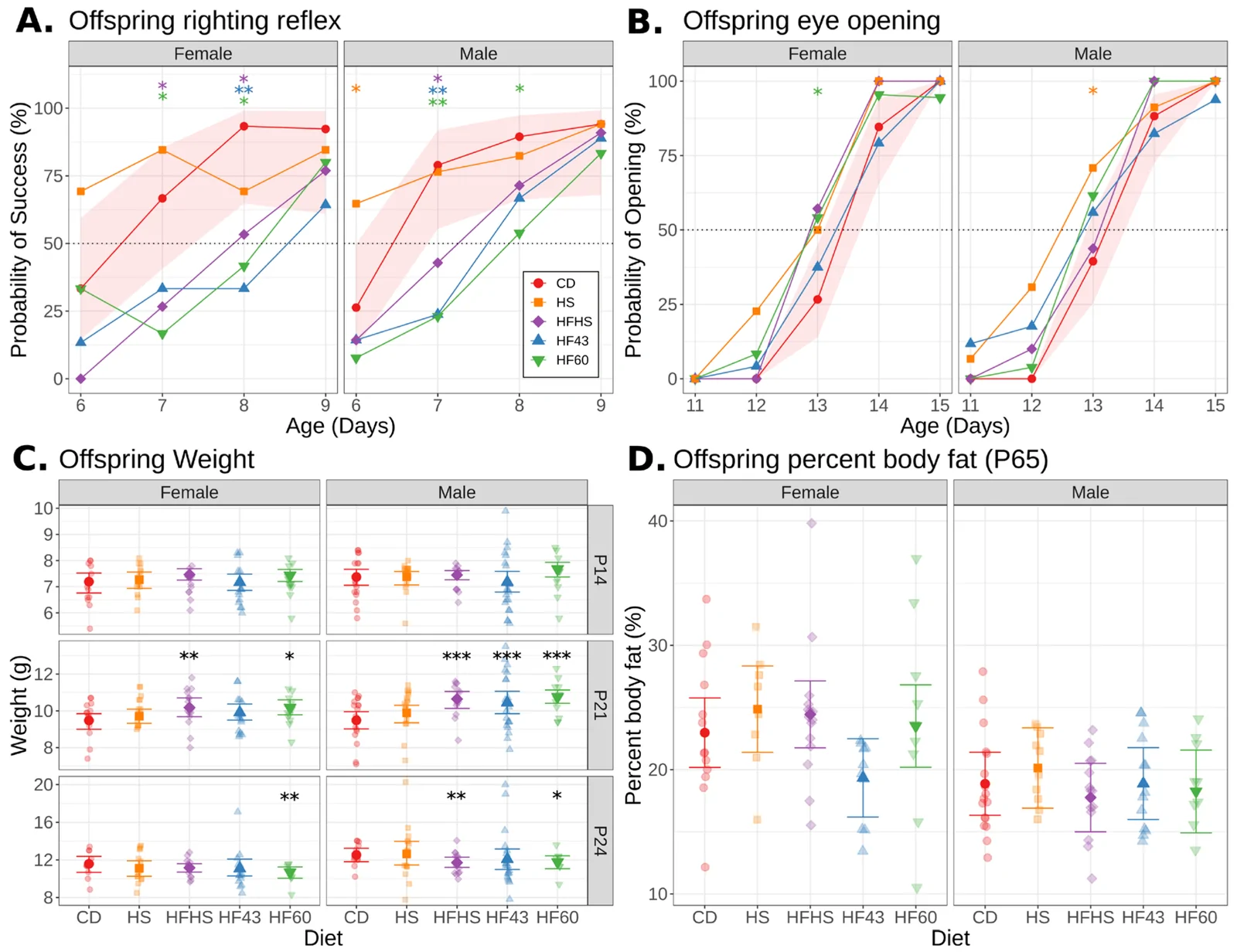
Offspring milestone and weight measures. (**A**) Offspring righting reflex milestone, with the data points representing average righting success of each diet group at each testing age. (**B**) Offspring eye-opening milestone, where the data points represent average eye-opening success (100% means both eyes opened) of each diet group at each measurement age. The ribbons in (**A**,**B**) represent the 95% confidence intervals generated from the outputs of their corresponding models, shown just for the CD group for visualization purposes. (**C**) Offspring weights at ages P14, P21, and P24. (**D**) The mean body fat percentage at P65 did not differ significantly between the diet groups. For (**C**,**D**), the mean and 95% confidence intervals plotted were generated from the outputs of their corresponding models, and the individual data points represent individual mice. The data points have been jittered horizontally for visualization purposes. Asterisks denote: * *p* < 0.05; ** *p* < 0.01; *** *p* < 0.001.

We evaluated the impact of diet on achievement of developmental milestones in offspring. Both the male and female offspring of the experimental diet groups with a high-fat component (i.e., HFHS, HF43, and HF60) showed delayed achievement of the righting reflex milestone, with a significantly lower probability of success (27.7% [11.3, 54.5]) on average at P7 compared to CD (72.8% [61.4, 84.2], *p* < 0.05; Figure 3A). The HS male offspring showed earlier achievement of the righting reflex milestone, with a higher success rate at P6 (CD 26.3% [11.4, 49.8] and HS 64.7% [40.4, 83.2], *p* < 0.05; Figure 3A). HS male and HF60 female offspring exhibited earlier eye opening, with a higher probability of eye opening at P13 compared to CD (males: CD 39.5% [25.4, 55.6] and HS 70.8% [50.2, 85.4], *p* < 0.05; females: CD 26.7% [13.9, 45.0] and HF60 54.2% [34.6, 72.5], *p* < 0.05; Figure 3B). The probability of eye opening in all other groups did not differ significantly from CD.

At weaning (P21), HFHS and HF60 female offspring were 10.2 g [9.9, 10.5]; both groups were heavier than CD female offspring (9.5 g [9.0, 9.9], *p* < 0.05; Figure 3C). Male offspring of all three high-fat diet groups weighed significantly more (HFHS 10.6 g [10.1, 11.1], HF43 10.4 g [9.8, 11.1], and HF60 10.8 g [10.4, 11.1]) than CD males (9.5 g [9.0, 10.0], *p* < 10^−4^; Figure 3C). However, weight differences were no longer present post-weaning at P24 (females: CD 11.6 g [10.8, 12.3], HS 11.1 g [10.3, 11.9], HFHS 11.2 g [10.7, 11.6], and HF43 11.1 g [10.3, 12.1], *p* > 0.07; males: CD 12.5 g [11.8, 13.3], HS 12.6 g [11.4, 14.1], and HF43 12.1 g [11.1, 13.2], *p* > 0.07; Figure 3C). Two experimental diet groups showed lighter weights than CD at P24 (female HF60 10.7 g [10.0, 11.2], male HFHS 11.7 g [11.2, 12.3] and HF60 11.8 g [11.1, 12.4], *p* < 0.05; Figure 3C). At P65, the diet groups containing a high fat content (i.e., HFHS, HF43, and HF60) showed modest weight decreases relative to CD (female avg 19.0 g [18.8, 19.2] compared to CD 19.7 g [19.3, 20.0]; male avg 24.2 g [24.0, 24.4] compared to CD 25.0 g [24.7, 25.3], *p* < 0.05). See Supplementary Figure S2 for the evolution of offspring body weight from P3 to adulthood. There was no significant difference in offspring body fat percentage at P65 across the diet groups for either sex (females: CD 23.0% [20.2, 25.8], HS 24.9% [21.4, 28.3], HFHS 24.4% [21.7, 27.1], HF43 19.3% [16.2, 22.5], and HF60 23.5% [20.2, 26.8], *p* > 0.05; males: CD 18.9% [16.3, 21.4], HS 20.1% [16.9, 23.4], HFHS 17.8% [15.0, 20.5], HF43 18.9% [16.0, 21.8], and HF60 18.2% [14.9, 21.6], *p* > 0.5; Figure 3D).

### 3.3. Perinatal Diet Alterations Modified Brain Structures in Adulthood

**Figure 4:**
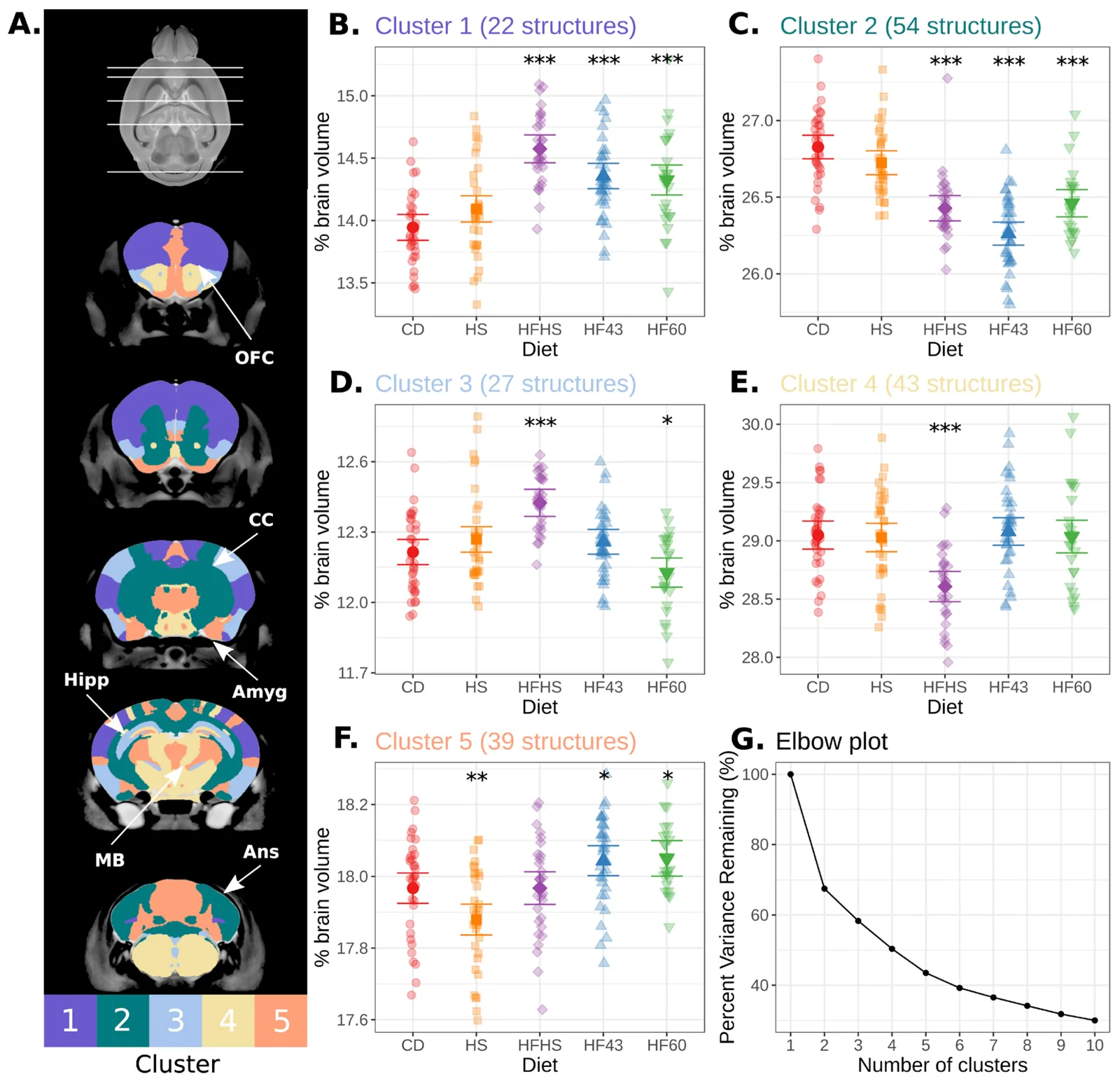
Cluster analysis showed groups of structures with a similar pattern of change across the offspring of diet groups high in fat content. (**A**) The structures were grouped into 5 clusters based on the impact of perinatal diet exposure. The top horizontal slice displays the location of the coronal cross-section slices. OFC: orbitofrontal cortex. CC: corpus callosum. Amyg: amygdala. Hipp: hippocampus. MB: midbrain. Ans: ansiform lobule. (**B**) Cluster 1 consisted of frontal, insular, and temporal cortical structures whose volumes were collectively larger in HFHS, HF43, and HF60 offspring. (**C**) Cluster 2 consisted of subcortical and cerebellar grey and white matter structures, whose volumes were collectively smaller in HFHS, HF43, and HF60 offspring. (**D**) Cluster 3 consisted of entorhinal and hippocampal cortical structures whose volumes were collectively larger in HFHS and smaller in HF60 offspring. (**E**) Cluster 4 consisted of subcortical grey and white matter structures and pituitary regions whose volumes were collectively smaller in HFHS offspring. (**F**) Cluster 5 consisted of subcortical grey matter and cingulate and entorhinal cortical structures whose volumes were collectively smaller in HS offspring and larger in HF43 and HF60 offspring. (**G**) The elbow plot of the percent variance remaining depending on the number of clusters used. In panels (**B**–**F**), the mean and 95% confidence intervals plotted were generated from the outputs of the linear model, and the individual data points represent individual mice after subtracting random effects of litter. The data points have been jittered horizontally for visualization purposes. Asterisks denote: * *p* < 0.05; ** *p* < 0.01; *** *p* < 0.001.

To evaluate spatial patterns of the effect of parental diet on offspring brain development, we first used cluster analysis to identify five groups of brain structures with similarities in volume change at P65 due to diet. Diets high in fat content had a similar impact on a subset of these clusters (Figure 4A). Cluster 1 mainly consisted of frontal, insular, and temporal cortical structures, and its collective relative volume was significantly increased in HFHS, HF43, and HF60 offspring by 4.5% [3.4, 5.6], 3.0% [1.9, 4.0], and 2.7% [1.6, 3.9], respectively, compared to the CD cluster relative volume of 13.9% [13.8, 14.0] (*p* < 10^−5^; Figure 4B). On the other hand, cluster 2 mainly consisted of subcortical and cerebellar grey and white matter structures whose collective volume was significantly decreased in HFHS, HF43, and HF60 offspring by 1.5% [1.0, 1.9], 2.1% [1.7, 2.5], and 1.4% [0.9, 1,8], respectively, compared to the original CD cluster relative volume of 26.8% [26.8, 26.9] (*p* < 10^−8^; Figure 4C). Although the high-fat-containing diet groups showed similar effects overall, HFHS offspring exhibited small but distinct differences in Clusters 3 and 4 (Figure 4D,E). The HS effect was minimal but most prominent in cluster 5 (Figure 4F). Supplementary Figure S3 shows cluster plots separated by sex. 

### 3.4. Diets High in Fat Content Rather than Sugar Content Affected Offspring Brain Structure

**Figure 5:**
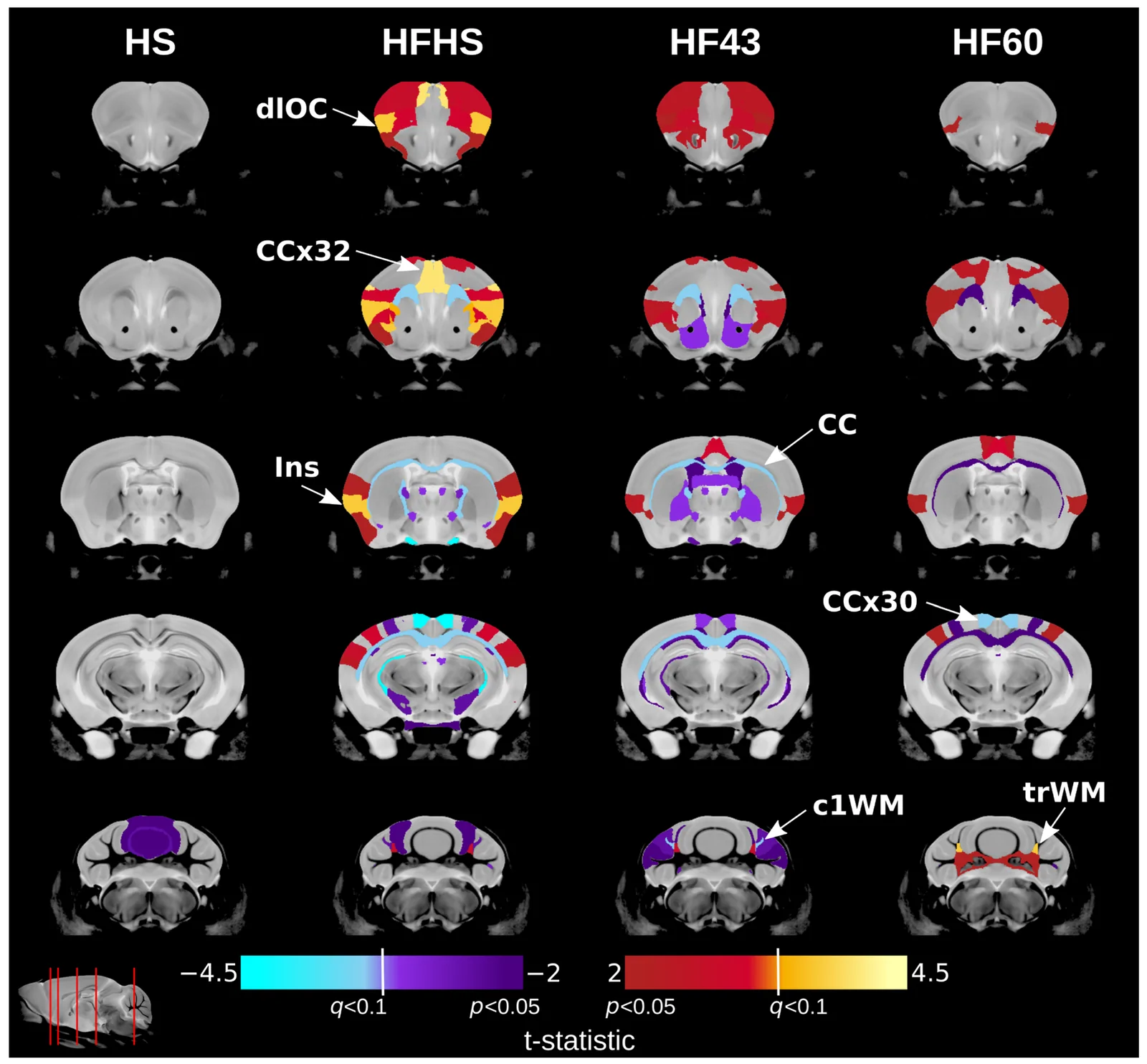
Perinatal diet exposure induces brain structure changes in offspring. Each row shows a coronal cross-section of the offspring brain with the diet groups across the columns. The slice indicator in the bottom left corner shows the location of the coronal slices (red lines). Warm colours indicate structures relatively larger in experimental diet groups and cool colours indicate structures relatively smaller in experimental diet groups as compared to the control diet group. Colours more extreme than the *q* < 0.1 threshold represent significant structures after correction for multiple comparisons across the diets at 10% FDR. Dark colours below the *q* < 0.1 threshold represent structures with an uncorrected *p*-value of less than 0.05. HS diet produces little change in offspring brains by adulthood. The other diets produce neuroanatomical changes with some similarities. dlOC: dorsolateral orbital cortex. CCx32: cingulate cortex area 32. Ins: insular region. CC: corpus callosum. CCx30: cingulate cortex area 30. c1WH: crus 1 white matter. trWM: trunk of simple and crus 1 white matter.

**Figure 6:**
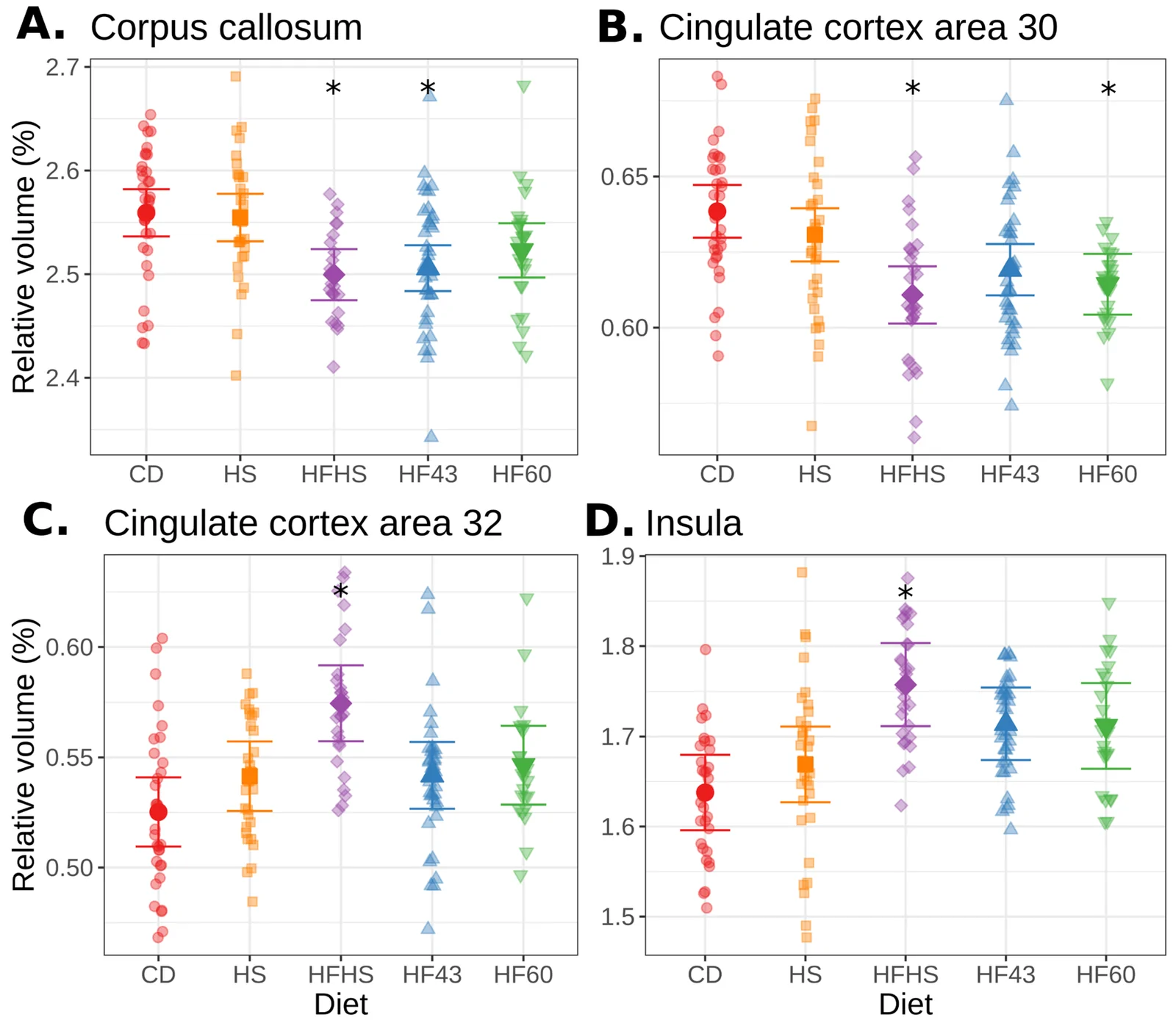
Plots of specific brain structures with volume changes induced by diets. (**A**) Corpus callosum was significantly smaller in HFHS and HF43 offspring. (**B**) Cingulate cortex area 30 was significantly smaller in HFHS and HF60 offspring. (**C**) Cingulate cortex area 32 and (**D**) insula were significantly larger in HFHS offspring. The mean and 95% confidence intervals plotted were generated from the outputs of the LME model, and the individual data points represent individual mice after subtracting random effects of litter. The data points have been jittered horizontally for visualization purposes. Asterisks denote: * *q* < 0.1.

We evaluated diet-induced changes across all individual structures in the brain atlas. Volumetric comparisons of each brain structure revealed that significant changes were seen in offspring exposed to diets with high fat content (Figure 5). After correcting for multiple comparisons, HF43 offspring showed an average −2.1% [−4.2, 0.05] change in relative corpus callosum volume from the baseline CD volume of 2.56% [2.54, 2.58] (*q* < 0.1; Figure 5 and Figure 6A) and significant volume decreases in two other white matter structures (*q* < 0.1; Figure 5). HF60 offspring showed a −3.8% [−5.9, −1.7] change in the relative volume of cingulate cortex area 30 relative to the CD value of 0.638% [0.630, 0.647] (*q* < 0.1; Figure 5 and Figure 6B) and volume changes in two other structures (*q* < 0.1; Figure 5). HFHS offspring showed significant changes in a greater number of structures (12 total, *q* < 0.1), including a relative volume decreases in the corpus callosum (−2.3% [−3.6, −1.0], *q* < 0.1; Figure 5 and Figure 6A) and cingulate cortex area 30 (−4.2% [−6.3, −2.3], *q* < 0.05; Figure 5 and Figure 6B) and volume increases in cingulate cortex area 32 (+9.5% [5.0, 13.8], *q* < 0.05; Figure 5 and Figure 6C) and the insular region (+7.3% [3.5, 11.1], *q* < 0.1; Figure 5 and Figure 6D). Interestingly, HS offspring did not show significant volume changes in individual structures compared to CD offspring after multiple comparisons correction. Of note, there were two structural changes that were common across diet groups: corpus callosum volume was decreased in both the HFHS and HF43 offspring, and cingulate cortex area 30 volume was decreased in both the HFHS and HF60 offspring. See Supplementary Table S3 for a complete listing. Structurewise analysis of diffusion imaging data showed overall limited differences between diet groups (Supplementary Figure S4 highlights mean diffusivity from the medial orbital cortex).

### 3.5. HFHS Offspring Phenotypes Correlate with Subgroups of ASD Mouse Model Phenotypes

**Figure 7:**
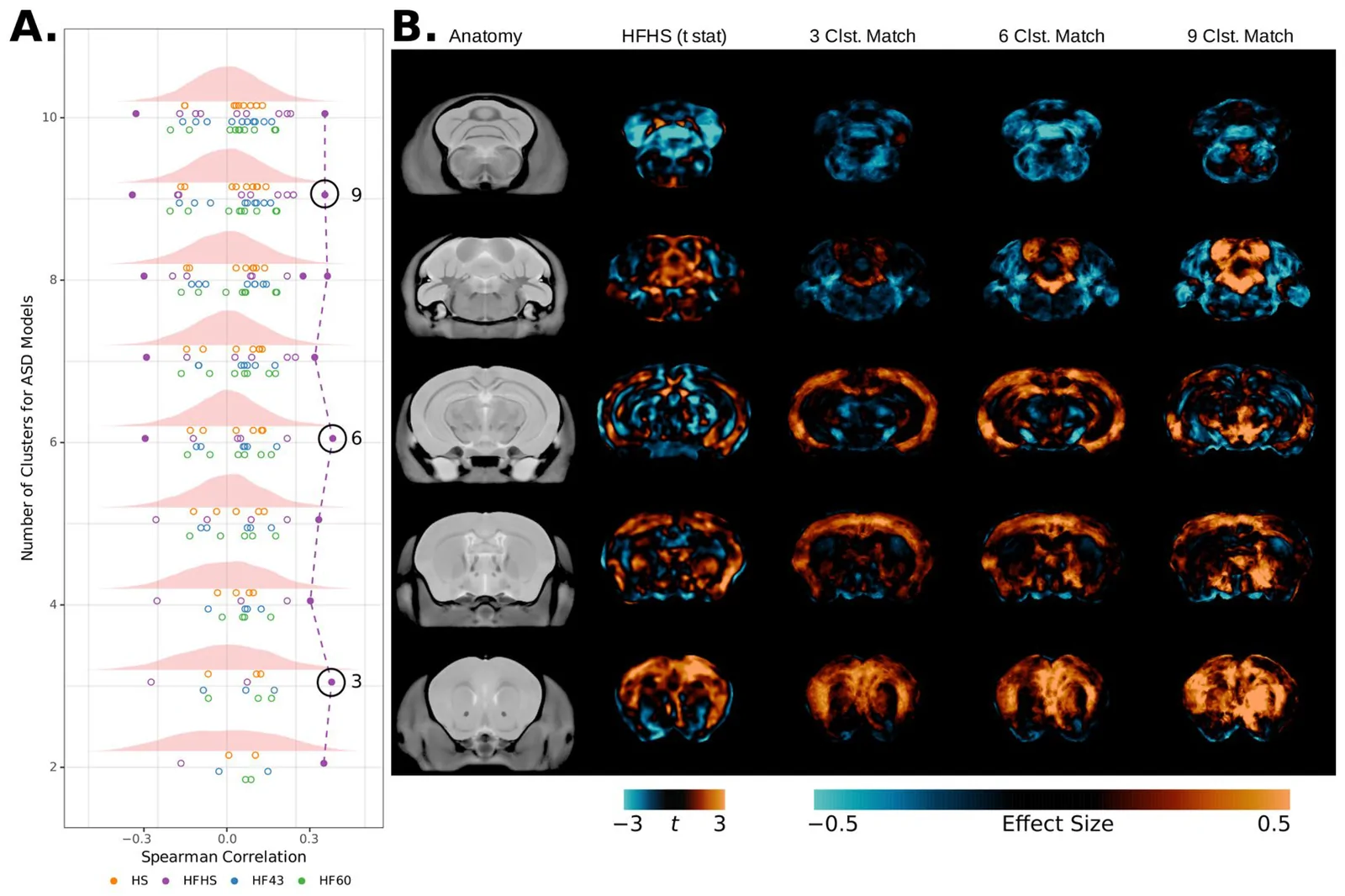
Comparison of diet-induced offspring brain phenotypes to models of ASD. Voxelwise *t*-statistic maps were generated summarizing the offspring brain phenotype for each parental diet. These were compared using a Spearman correlation across voxels to effect size maps generated from a large study of 135 ASD and ASD-related mouse models, which were clustered into groups of related models. (**A**) Results of the correlation are plotted for each diet for the 2 to 10 cluster solutions of the ASD mouse models. The red distribution represents an empirical null distribution generated by permutation of all diet labels. The points indicate measured correlations with each cluster group coloured by diet, with filled points indicating *p* < 0.05 based on the permutation. The purple dashed line highlights that the HFHS offspring showed a significant degree of correlation to at least one group across all cluster solutions and consistently exhibited higher correlation than the other diet groups. (**B**) Voxelwise *t*-statistic maps are shown for the HFHS group with comparison to the highest correlation match achieved for the 3, 6, and 9 cluster ASD model solutions, which correspond to the circled points along the dashed line in (**A**).

We empirically evaluated the relevance of the diet-induced offspring phenotypes to the panel of mouse ASD models described in Ellegood et al. [43]. The neuroanatomical phenotypes in the HFHS-diet offspring, in particular, showed a high degree of correlation with at least one ASD model group across all of the cluster solutions generated in the reference panel of ASD models (Figure 7A; with 2 through 10 cluster solutions explored). Spatial maps in Figure 7B display the high-correlation matches for visual comparison. The HFHS matches from the ASD models were previously associated with processes of chromatin organization, chromatin modifying enzymes, histone methylation and/or transcriptional regulation [43].

## 4. Discussion

Our data demonstrate that perinatal diet exposure has a lasting impact on brain structure despite similar mothering behaviour in early life and resolution of initial impacts on weight and body composition in offspring. The high-fat-only and high-fat/high-sugar combined parental diets, but not the high-sugar-only diet, affected offspring’s brains in early adulthood, indicating that high fat is a key parental diet factor influencing offspring brain development.

Parental obesity and metabolic status are known to be important risk factors for ongoing childhood obesity, especially since perinatal environmental exposures generally continue into childhood [44,45,46]. Ongoing exposures in childhood are also likely to affect the brain. Thus, in our study, we sought to isolate the impacts of perinatal diet exposure by placing pups on CD after weaning until imaging in early adulthood. Although, based on our data, we cannot rule out the possibility of protracted influences from diet-induced adipokines, interleukins, or insulin regulation, our data demonstrate persistent brain changes despite normalized body weights and fat composition, suggesting that perinatal diet exposures lead to long-lasting changes in the brain, consistent with previous findings [15,17,47].

Due to the association between pre-pregnancy body mass index and postpartum depression [48,49] and the link between maternal care behaviours and offspring neurodevelopment [50,51], we assessed the impact of different diets on maternal behaviour. Although our analysis indicated changes in maternal behaviour due to experimental diets, we consider these to be subtle and not a strong indicator of alterations in mothering activity.

We also explored whether perinatal diet induced early-life developmental changes in offspring. The performed assays assessed developmental neural function in young rodents. In mice, righting reflex success requires proper muscle and lower motor neuron function, spinocerebellar function, sensory function, and arousal [52]; eye opening is a milestone that reflects physical development [53]. Our data did reveal small changes in early-life milestones dependent on perinatal diet exposure, indicating that, in addition to behavioural changes previously reported in adult offspring [15,54,55], perinatal diet can lead to changes in early-life neurodevelopment. Additional studies should also assess brain structure in the early postnatal period.

Specific brain structures whose relative volumes were altered in the offspring of the experimental diet groups have previously been reported to be affected in human NDD populations. The cingulate cortex, which was altered both in HFHS and HF60 offspring in our data, plays an important role in social cognition and reward-related processing [56]. Volume abnormalities in this region have been reported in humans with ASD [57,58], ADHD [59], and intellectual disabilities [60]. This structure may be implicated in impairments in social learning [61], aberrant reward processing [62], and cognitive deficits [63]. The corpus callosum was altered both in HFHS and HF43 offspring. It has been reported to be altered in volume and connectivity in ASD [64,65,66] and ADHD [67,68,69] in human populations and associated with severity of symptoms.

Diets high in fat content led to changes in offspring brain structure in early adulthood, while the high-sugar diet did not. This observation may provide insights into the mechanisms by which diet affects the developing brain, as fat [70,71] and cholesterol [72,73] play important roles in brain development, with particular significance for the structure and function of neurons and oligodendrocytes [74]. However, we note that the diet combining high-fat and high-sugar (i.e., HFHS) produced a greater number of significantly altered structure volumes than diets high in fat content only, so that effects of high sugar cannot be entirely ruled out [75,76]. Moreover, the HFHS effects best matched a subset of phenotypes from a panel of ASD-related mouse models, which suggests that processes of chromatin organization, chromatin modifying enzymes, histone methylation and/or transcriptional regulation [43] should be further explored for their role in the neurodevelopment of HFHS-exposed offspring. We also note that the modified diets necessarily resulted in changes in total protein and/or carbohydrate content. While our study design primarily focused on probing changes in fat and sugar content, it would be valuable to further explore how these macronutrient changes contribute to the observed offspring brain structure phenotypes. 

Future studies could also examine diet-related molecular pathways—such as insulin signalling [77], inflammatory markers [78,79], and immune modulations [79,80]—alongside more comprehensive characterization of peripheral metabolic outcomes [81] to establish a clearer connection to potential mechanisms underlying the observed structural changes. For example, we chose to measure dam glucose tolerance and body composition in a separate cohort of mice. Performing these measurements in the same group would enable investigation of links between offspring brain structure outcomes and degree of maternal metabolic dysregulation. It would also be of interest to explore the genetic vulnerabilities identified from human data in the context of the mouse model, which may show greater or lesser sensitivity to diet effects [82,83]. It is also intriguing that the impacts of the HF60 diet did not differ substantially from those of the HF43 diet, raising the possibility of a ceiling effect of dietary fat content which could be explored using a panel of additional diets. Finally, detailed behavioural studies could be done to assess the functional impact of the observed structural changes [84,85,86], such as behavioural alterations related to ASD phenotype. Our focus was to dissect the different components of the diets, and now, based on our data, future studies could examine the behavioural impacts of the diets that were shown to have a more significant impact on brain structure.

## 5. Conclusions

In summary, we investigated the effects of fat and sugar on offspring brain structure in a mouse model to improve understanding of how parental diet impacts brain development. High-fat and combined high-fat/high-sugar diets affected offspring brain structure in adulthood, but diets high in simple sugar did not. These findings suggest a mechanism primarily dependent on high fat consumption (though sugar may have an influence when consumed with high fat content). Brain structures including the corpus callosum and cingulate cortex, with structural and functional relevance to human NDD populations and phenotypes, were altered in offspring after perinatal diet exposure. Although interspecies differences must be considered, these findings highlight the importance of parental diet before and during pregnancy and provide insight into mechanisms by which perinatal diet may affect NDD risk.

## Figures and Tables

**Table 1:** Dietary macronutrient content summary.

Diet	Diet ID ^1^	Caloric Density (kcal/g)	kcal (%)			Sucrose Content (% Weight)
			Fat	Protein	Carbohydrate	
CD	TD08485	3.6	13	19	68	12
HS	TD05230	3.7	12	19	69	34
HFHS	TD88137	4.5	42	15	43	34
HF43	TD210336	4.5	43	15	42	12
HF60	TD210337	5.2	60	16	24	12

^1^ Inotiv, formerly Envigo, Teklad Diets, IN, USA.

## Data Availability

Data collected for this project may be accessed through the Zenodo data repository at https://doi.org/10.5281/zenodo.23348707.

## Supplementary Materials

The following supporting information can be downloaded at https://www.mdpi.com/article/10.3390/nu18213539/s1: Supplementary Methods; Supplementary Results; Table S1: Additional detail on husbandry standards; Table S2: Additional diet information; Table S3: Percent change in relative structure volumes from the effects of diet, sex, and their interaction; Figure S1: Offspring milk spot; Figure S2: Offspring weight gain from birth to adulthood; Figure S3: Cluster groupings separated by sex; Figure S4: Results for mean diffusivity derived from diffusion tensor imaging for the medial orbital cortex. Reference [87] is cited in the supplementary materials.

## Abbreviations

The following abbreviations are used in this manuscript:

NDD	Neurodevelopmental disorder
MRI	Magnetic resonance imaging
ASD	Autism spectrum disorder
ADHD	Attention-deficit/hyperactivity disorder
TCP	The Centre for Phenogenomics
CD	Control diet
HS	High-sugar diet
HF43	High-fat diet (43% of kcal from fat)
HF60	High-fat diet (60% of kcal from fat)
HFHS	High-fat/high-sugar diet
AUP	Animal Use Protocol
P	Postnatal day
MNI	Montreal Neurological Institute
MAGeT	Multiple automatically generated templates
LME	Linear mixed effects
