# Supplementary material for: Disentangling Fat and Sugar in the Western Diet: Parental Consumption of a High-Fat Diet Alters Offspring Brain Development
Source: Nutrients. 2026 Oct 27;18(21):3539. doi: 10.3390/nu18213539 (PMC13765270; doi:10.3390/nu18213539)
Supplement: Supplementary file 1 [file nutrients-18-03539-s001.zip › nutrients-4576133-supplementary.pdf]

Supplementary Materials

Supplementary Methods

Diffusion tensor imaging (DTI) was performed with a 3D spin-echo echo planar imaging sequence, running 6 b0 scans and 30 diffusion directions, with the following parameters: TR = 300 ms, TE = 44 ms, 12 echos, effective Bval = 2000 s/mm^2, field-of-view = 25.6 x×19.2 × 19.2 mm, matrix size = 320 × 240 × 240, segmented 20 times, 1 average, total acquisition time = ~14.5 h, 80 μm isotropic resolution.

For DTI processing, all diffusion-weighted images were registered together and then mapped to the anatomical images for quantitation. For this purpose, the b0 images and diffusion direction images for each sample were separately registered using ANTs to produce b0 average and diffusion average images using the image with minimum entropy as the target. The b0 and diffusion average images were then registered together, and transformations were applied to align all images in the average b0 space. To account for signal intensity variations unrelated to diffusion over the course of the long overnight scans, median whole-brain intensities across the data set were used for normalization. To generate DTI maps (i.e., fractional anisotropy and mean diffusivity), log intensity data were modeled using convex optimization (cvxopt, version 1.2.5) [87] with iterative reweighting used to minimize effects of outliers. The resulting DTI maps were then resampled to map onto the MAGeT-segmented anatomical images (described in the main text) for segmentation of brain structures and subsequent structure-wise analysis of the DTI metrics.

Fractional anisotropy (FA) and mean diffusivity (MD) measures of each brain structure were fit with a LME model. Fixed effects were included for diet (categorical), sex (categorical), and their interactions. A covariate for diffusion image intensities (z-scored) was included as a

possible confound and a random effect was used to account for litter. Multiple comparisons were handled as described in the main text.

Supplementary Results

Diffusion tensor imaging overall showed no significant differences between diet groups. There was no difference in FA across the diet groups. A small statistically significant change in MD was observed in the medial orbital cortex for females but attributed to elevated MD measurements in the CD group (see Supplementary Figure 4).

Elements of husbandry	The Centre for Phenogenomics animal facility
Animal facility geographic location	GPS coordinates (43° 39' 28.872" N, 79° 23' 31.884" W); altitude of 100.6 m
Animal room temperature	21-23 °C (monitored by a building automation system)
Animal room relative humidity	40-60% (monitored by a building automation system)
Animal room air changes	15-20 air changes per hour
Animal room lighting	12-hour light/12-hour dark cycle (lights on at 7am, lights off at 7pm); controlled by building automation system; animal lighting provides 30 foot-candles average illumination at 3 feet above floor level; staff work lighting provides 60-70 foot-candles average illumination at 3 feet above floor level
Contact bedding	¼ inch corn cob; Anderson's
Nesting material	Enviropak (pouch containing Envirodri), North American Lab Supply
Caging	Greenline Individual Ventilated Caging (IVC), Greenline GM500 cage model, Tecniplast; cage floor area of 501 cm^2
Cage-change frequency	Every 14 days
Social environment	2-5 mice in each cage; pregnant mice singly housed
Drinking water	Avidity Science, recirculating Automated watering system; reversed osmosis (R/O) water, acidified, ultraviolet light filtration
Animal identification method	Ear notch for parent mice; paw tattoo for offspring mice
Environmental enrichment	For singly housed mice, wood gnawing blocks (Bio-Serv #K3511) and bio-tunnels for mice (Bio-Serv #3556-400)
Disinfectants used in the animal facility	Clidox, Pharmacal
Methods used to handle mice	Hold by the base of the tail when transferring (with minimal time held in the air), scruff restraint for injections
Sex of the experimenter	Female
Microbiologic status of the facility	Refer to the legend below

Supplementary Table S1. Additional detail on husbandry standards.

Husbandry standards at the Centre for Phenogenomics animal facility. Viruses excluded in the facility: Mouse parvoviruses (MVM/MPV 1-5), Mouse coronavirus (MHV), Murine rotavirus (MRV/EDIM), Mouse theilovirus (TMEV, GDVII), Adenovirus type 1 & 2 (MAV-1 & MAV-2), Reovirus types 1-4, Pneumonia virus of mice, Sendai, Ectromelia (mousepox), and Lymphocytic choriomeningitis virus. Bacteria excluded in the facility: Citrobacter rodentium, Mycoplasma pulmonis, Streptobacillus moniliformis, Clostridum piliforme, CAR bacillus, Pseudomonas aeruginosa, Salmonella, Bordetella bronchiseptica, Bordatella pseudohinzii, Campylobacter, Corynebacterium kutscheri, Corynebacterium bovis, Staphylococcus aureus, Streptococcus pneumonia, Klebsiella pneumoniae, Klebsiella oxytoca, and Beta hemolytic Streptococcus group

A, B, C, G. Parasites/Protozoa/Fungi excluded in the facility: Fur mites (Myobia, Myocoptes, Radfordia), Pinworms (Aspiculuris, Syphacia), Giardia, Spironucleus muris, Cryptosporidium, and Pneumocystis.

Component (g/kg)	CD	HS	HFHS	HF43	HF60
Casein	195	195	195	195	231
Sucrose	120	341	341	120	120
Corn starch	433	212	150	271	34.3
Anhydrous milkfat	37.2	37.2	210	210	344
Cellulose	50	50	50	50	50
Mineral mix	35	35	35	35	42
Calcium carbonate	4	4	4	4	4
Vitamin mix	10	10	10	10	10
Ethoxyquin (antioxidant)	0.01	0.01	0.04	0.04	0.07

Supplementary Table S2. Additional diet information.

Structure	Percent brain volume	SE	Percent change								
			HS	HFHS	HF43	HF60	Sex	HS: Sex	HFHS: Sex	HF43: Sex	HF60: Sex
lobule 9 white matter	0.059	0.001	-1.8	-5.2	-4.3	-2.8	2.2	-1.4	-2.6	2.0	-1.4
Primary somatosensory cortex dysgranular zone	0.081	0.001	0.0	-4.5	-2.5	-5.7	0.3	-0.4	-3.3	-4.4	-4.5
Cingulate cortex area 30	0.638	0.004	-1.2	-4.3	-3.0	-3.8	-2.6	0.1	1.1	0.5	2.1
optic tract	0.330	0.003	0.7	-5.0	-2.8	-1.6	-0.1	0.4	2.3	3.3	3.6
crus 1 white matter	0.081	0.001	0.0	-3.5	-4.1	0.0	-0.7	-0.2	0.0	2.4	2.5
stria terminalis	0.194	0.001	-0.9	-2.3	-2.6	-0.9	-0.9	0.4	2.2	2.9	4.2
internal capsule	0.634	0.003	-0.7	-2.7	-2.3	-1.0	0.8	-1.1	-0.7	0.6	0.6
corpus callosum	2.559	0.012	-0.2	-2.3	-2.1	-1.4	1.2	-1.1	-0.6	0.8	1.4
bed nucleus of stria terminalis	0.299	0.002	-0.4	0.5	-0.5	-0.2	8.6	-0.5	-1.6	0.6	1.2
Medial amygdala	0.240	0.001	1.3	0.4	0.4	0.1	8.7	1.3	-1.9	-0.3	1.6
Accessory olfactory bulb granule cell layer	0.054	0.001	-1.4	3.6	0.7	-0.2	7.6	-4.0	-3.9	-3.5	-4.9
Primary somatosensory cortex upper lip region	1.213	0.011	-0.1	2.2	0.7	0.6	-4.8	5.4	0.6	-0.9	-1.4
Frontal association cortex	1.652	0.015	1.0	3.7	3.0	1.2	-5.3	2.0	1.8	-1.2	-2.3
Dorsal nucleus of the endopiriform	0.320	0.004	1.9	6.0	3.3	1.4	-0.4	0.2	-0.7	-2.0	-0.4
Frontal cortex area 3	0.163	0.002	0.9	5.6	2.9	3.3	-2.4	-0.6	2.5	0.6	0.7
Claustrum	0.067	0.001	0.3	7.0	3.3	3.8	-3.1	-1.6	3.9	3.9	5.0
Insular region not subdivided	1.638	0.021	1.9	7.3	4.7	4.5	-2.7	0.7	2.8	0.0	1.8

trunk of simple and crus 1 white matter	0.033	0.000	0.3	5.5	5.5	8.0	-4.4	3.8	5.8	5.3	-2.6
Dorsolateral orbital cortex	0.189	0.003	2.1	8.9	5.1	3.4	-5.4	3.3	0.5	0.2	6.3
Cingulate cortex area 32	0.525	0.008	3.1	9.4	3.2	4.0	-0.3	-2.6	3.5	-2.8	-4.6
Claustrum ventral part	0.132	0.003	5.7	13.4	6.3	7.0	1.7	-6.7	-3.1	-3.9	2.0

Supplementary Table S3. Percent change in relative structure volumes from the effects of diet, sex, and their interaction.

Percent brain volume column represent the baseline volume of the structure relative to the whole brain (i.e., relative volume), for CD mice with sexes combined. SE column represent the standard error of the percent brain volume of the structures. Percent change columns indicate percent change in the relative volume of the structures, colour-coded with red for increasing and blue for decreasing. HF43, HF60, HFHS, and HS columns represent the effect of the respective diets with males and females combined. Sex column represents the offset of CD males from the CD female baseline. HF43:Sex, HF60:Sex, HFHS:Sex, and HS:Sex columns indicate the interaction effect of the respective diets with male sex. The cells filled in with gray represent a significant change after 10% FDR (i.e., q<0.1).

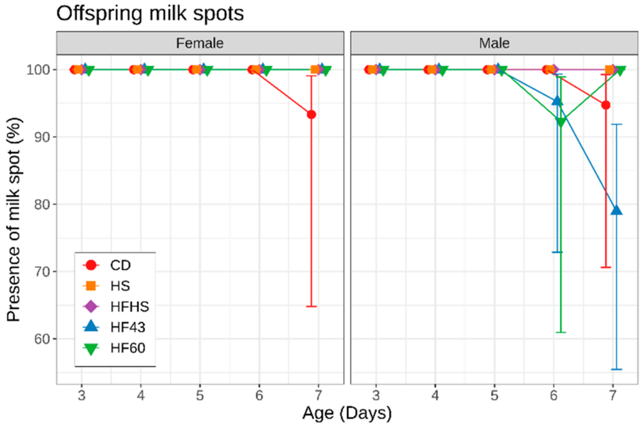

Supplementary Figure S1. Offspring milk spot

The percentage of milk spot present in the offspring did not differ significantly between diet groups. Females at P7 ($p<0.9$): CD 93.3% [64.8, 99.1], HF43 100.0% [100,100], HF60 100.0% [100,100], HFHS 100.0% [100,100], and HS 100.0% [100,100]. Males at P7 ($p>0.1$): CD 94.7% [70.6, 99.3], HF43 78.9% [55.4, 91.9], HF60 100.0% [100,100], HFHS 100.0% [100,100], and HS 100.0% [100,100]. The data points have been jittered horizontally for visualization purposes.

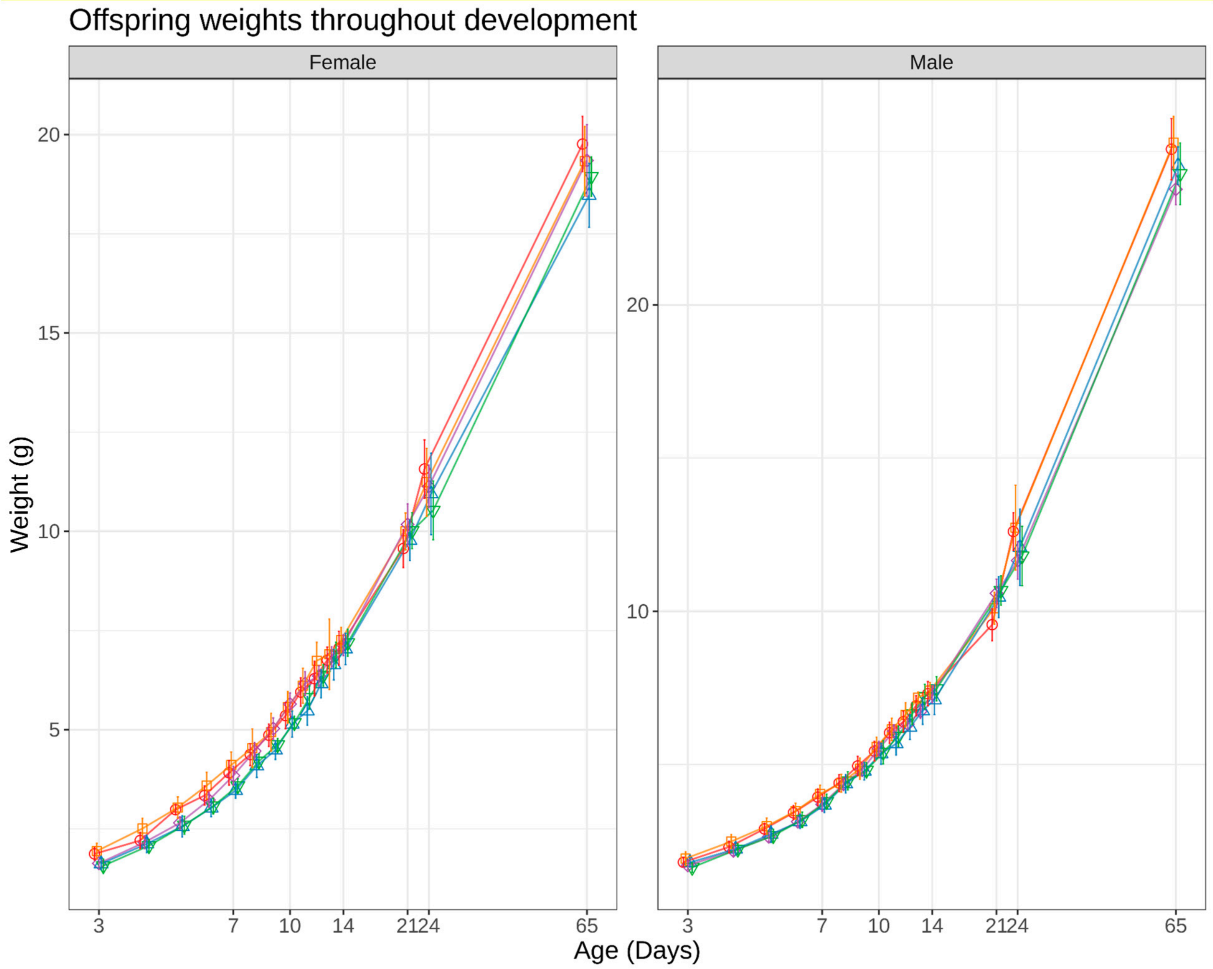

Supplementary Figure S2. Offspring weight gain from birth to adulthood

The evolution of offspring body weight from P3 to adulthood. A logarithmic x-axis was used for a more equal spacing between the timepoints. All error bars represent 95% confidence intervals, calculated by bootstrapping 500 times with replacement. The data points have been jittered horizontally for visualization purposes.

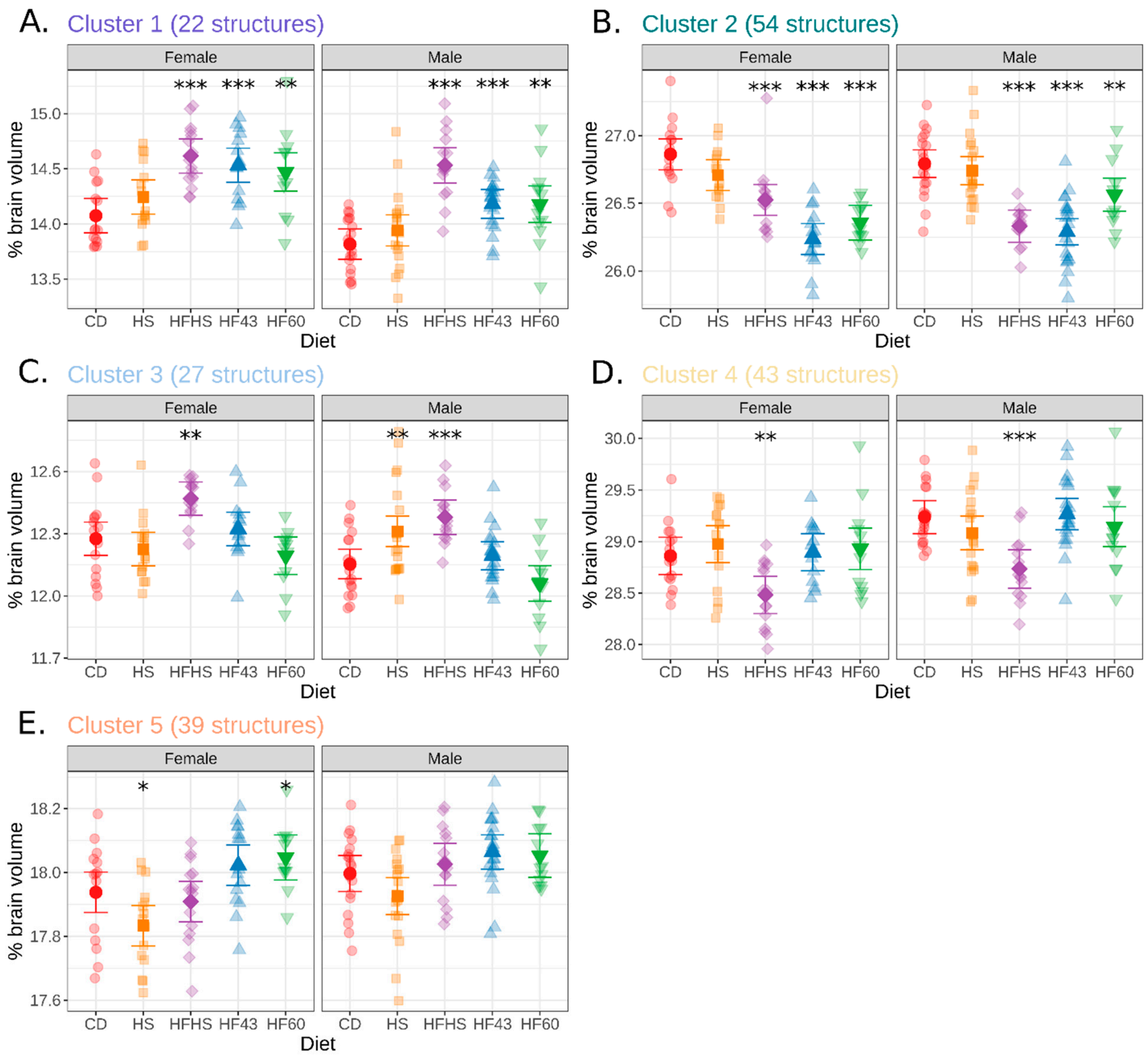

Supplementary Figure S3. Cluster groupings separated by sex

Cluster 1 **(A)** and Cluster 2 **(B)** showed the same patterns of volume change in the diet groups across female and male. **C** Cluster 3 showed slightly different patterns across female and male, such that HS group shows increased volume only in males. **D** Cluster 4 showed the same pattern of volume change across female and male. **E** Cluster 5 showed slightly different patterns across female and male, such that HF60 and HS are significantly changed in collective volume only in females. The mean and 95% confidence intervals plotted were generated from the outputs of the linear model, and the individual data points represent individual mice after subtracting random effects of litter. The data points have been jittered horizontally for visualization purposes. Asterisks denote: *p<0.05; **p<0.01; ***p<0.001.

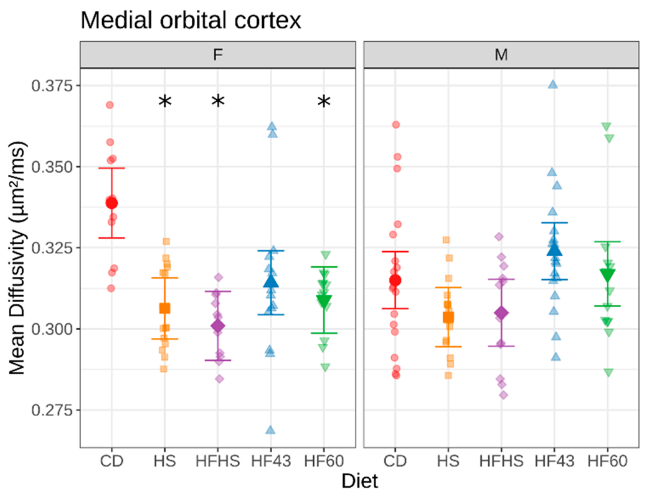

Supplementary Figure S4. Results for mean diffusivity derived from diffusion tensor imaging for the medial orbital cortex

There was a significant difference in only the mean diffusivity in the medial orbital cortex of HS, HFHS, and HF60 group offspring relative to CD in females after 10% FDR. The mean and 95% confidence intervals plotted were generated from the outputs of the linear model, and the individual data points represent individual mice after subtracting random effects of litter. The data points have been jittered horizontally for visualization purposes. Asterisks denote: *q<0.1.
